# The climate benefit of a greener blue hydrogen

**DOI:** 10.1038/s41598-025-18765-6

**Published:** 2025-09-29

**Authors:** Didier Hauglustaine

**Affiliations:** Laboratoire des Sciences du Climat et de l’Environnement (LSCE), CEA- CNRS-UVSQ, Gif-sur-Yvette, France

**Keywords:** Climate sciences, Environmental sciences, Energy science and technology

## Abstract

Previous studies have demonstrated the potential benefit of a future hydrogen economy in terms of reducing CO_2_ emissions. The hydrogen leakage rate and the green hydrogen fraction in the mix were identified as key factors in maximising the climate benefit of this energy transition. This study highlights the importance of blue hydrogen production hypotheses for a climate-beneficial transition to a hydrogen economy. The benefits are substantial when blue hydrogen is produced properly using an efficient CO₂ sequestration hydrogen production plant and minimizing the rate of upstream CH₄ leakage. The rate of hydrogen leakage remains an important parameter to consider throughout the entire value chain. Based on various scenarios of the development of a 21st century hydrogen economy, we estimate significant CO₂ emission reductions of 266–418 GtCO₂eq (up to 395–675 GtCO_2_eq in the case of a “high hydrogen demand” scenario) between 2030 and 2100. This cumulative reduction in CO₂ emissions translates into a reduction in global warming of 0.12–0.19 °C (0.18–0.30 °C for a “high hydrogen demand”) by the end of the century.

**Supplementary Information:**

The online version contains supplementary material available at 10.1038/s41598-025-18765-6.

## Introduction

The use of molecular hydrogen (H_2_) as a clean energy transition fuel is receiving increasing attention, and is often being presented as a key element in disrupting the future energy value chain^1–5^. According to some scenarios, hydrogen could account for up to 22% of the final energy consumption in 2050^6^. In particular, H_2_ is considered a potential future energy storage vector, for use in hard-to-abate activity sectors, such as heavy industry, long-distance transportation, as well as for domestic use when blended with natural gas. Meeting the climate objectives of the Paris Agreement^7^ and achieving net zero emissions by 2050 requires the large-scale deployment of clean energy technologies. Low-emission hydrogen, ammonia, and hydrogen-based synthetic fuels could therefore play an important role in this decarbonisation and in the integration of renewable energy into the energy system. The cost of clean hydrogen seems to prohibit it from providing more than 9% of the final energy demand^8^ but there is significant variance across the different models regarding the role of H_2_ in future deep decarbonisation^9^. However, it is still assumed that the long-term outlook for clean hydrogen is strong, despite the challenging near-term environment caused by recent cost increases, regulatory uncertainty, and the necessary filtering out of less competitive projects^10^.

Leakage of hydrogen is an issue and fugitive emissions of this probe-to-leak gas have been identified as important not only for safety resasons, but also due to their climate impact^11–14^. It is recognised that hydrogen indirectly contributes to global warming by affecting the lifetime and concentration of greenhouse gases through its involvement in atmospheric chemistry. In particular, the tropospheric oxidation of H_2_ depletes the hydroxyl radical (OH). Since OH is the main sink for methane, a potent greenhouse gas, this results in the atmospheric lifetime of methane being lengthened. H_2_ and methane are also precursors of tropospheric ozone and a photochemical source of water vapour in the dry stratosphere, and both of these also act as greenhouse gases. While recent studies suggest that fugitive emissions could be of minor importance in some sectors of hydrogen use^15^ leakage could still be significant in long-distance pipeline transport or in specific sectors, such as liquid hydrogen production and use.

In addition to these fugitive emissions of hydrogen impacting the climate, the potential of a future hydrogen economy to mitigate climate change is largely determined by the greenhouse gas emission intensity of the hydrogen production method and the related emissions from the feedstock^11,15–17^. Emissions intensities vary widely among hydrogen production routes. Low-carbon hydrogen can be produced using energy resources such as solar, geothermal, wind, biomass, nuclear, or from fossil fuels accompanied by Carbon emission Capture and Sequestration (CCS). In the European Union, the Renewable Energy Directive (RED III) sets a threshold of 3.38 kgCO_2_eq/kgH_2_ for low-carbon hydrogen^18^. Green hydrogen production based on renewable energy emits less than 2 kgCO₂eq/kgH₂^19^, and as little as 0.8–0.9 kgCO₂eq/kgH₂ by 2050, according to the International Energy Agency’s Net Zero Emissions future scenario^20^. This makes green hydrogen a highly appealing candidate for implementation in a future decarbonised economy.

Today, the demand for 94 Mt/yr of hydrogen is almost entirely met by production from unabated fossil fuels and by-product hydrogen from industrial processes that also use fossil fuels as feedstock, resulting in an emission intensity in the range of 12–13 kg CO_2_eq/kgH_2_^3^. This leads to more than 900 Mt/yr of direct CO_2_ emissions^3^. Currently, the production of low-emission hydrogen is less than 1 Mt/yr, almost all of which comes from fossil fuels with CCS with only 35 kt/yr of H_2_ produced from electrivity via water electrolysis^3^. There are numerous projects worldwide aimed at scaling up low-carbon hydrogen production processes^2,3,5^. However, clean hydrogen production methods face significant technological and environmental challenges. For instance, the carbon footprint of hydrogen produced by electrolysis using renewable or nuclear energy varies significantly depending on the energy source: production using wind energy results in the lowest emissions, whereas production using solar energy results in higher emissions^16,17,21^. Furthermore, to reduce emissions, it is crucial to prevent grid-connected electrolysers from increasing fossil-based electricity generation.

In the case of hydrogen production through steam methane reforming of natural gas combined with CCS, often referred to as blue hydrogen, the efficiency of the carbon sequestration technology^22,23^ and the intensity of upstream fugitive emissions associated with the extraction, the compression, the liquefaction, and transport of the feedstock are crucial parameters^24,25^. The oil and gas industry strongly supports the blue hydrogen production from natural gas because it enables the production of clean fuels using existing gas production, distribution, and storage facilities. Many argue that blue hydrogen is therefore essential for developing a long-term market for hydrogen. From a climate perspective, the issue with blue hydrogen is that it depends on CCS technologies and on natural gas which is susceptible to leakage. Commercially viable carbon dioxide capture remains an aspiration and carbon capture can never be 100% efficient. Furthermore, the climate impact of upstream methane leakage is highly uncertain^26^ and recent studies have therefore emphasised the importance of properly producing blue hydrogen^27^. Current blue hydrogen power plants have a significant climate impact when their entire lifecycle is considered^22^. However, these figures have been disputed, with the argument being made that significant improvements in carbon sequestration rates could be achieved in the future by theoretical power plants under investigation^28–30^. Another proposed alternative for low-carbon pathway is the production of hydrogen from the pyrolysis of methane driven by renewable energy. This process involves the co-production of solid carbon black, for which there is an existing market^31^.

In this study, we examine the sensitivity of the climate benefit of a hydrogen economy to the carbon intensity of blue hydrogen production using recent hydrogen climate metrics. Specifically, we demonstrate the impact of different assumptions about blue hydrogen production on the reduction of cumulative CO₂ emissions in relation to the long-term climate goal set out in the Paris Agreement^7^.

## Results

### The carbon footprint of blue hydrogen production and use

The methodology used in this study to determine the climate impact of a developing future hydrogen economy worldwide is based on our previous study^11^. The various emission metrics (e.g., GWP, GTP, and CGTP) required to calculate the CO_2_ equivalent emissions of H_2_ and CH_4_ fugitive emissions were derived directly from our previous calculations^11^. These emission metrics align with those of a recent model intercomparison^12^, and are used in this study to enable comparison with our previous estimates and to emphasize the climate impact resulting from various assumptions about blue hydrogen production. The methodology is based on calculating the equivalent CO_2_ emissions from replacing fossil fuels with hydrogen fuel, which reduces fossil fuel CO_2_ emissions (See “Methods” and Supplementary Table S1 for the metrics used in this study).

In our previous calculations of the climate impact of hydrogen^11^, we based our analysis on CO₂ emissions during the steam methane reforming (SMR) process to drive the SMR itself, the amount of methane required as a feedstock upstream, and the CO₂ capture rates in the case of blue hydrogen, for which we used specific estimates^22^. In our current analysis, we investigate how sensitive the climate impact of hydrogen is to these assumptions, considering different cases for blue hydrogen production. Five cases are considered for blue and turquoise hydrogen production plants. Table 1 summarises and compares the key assumptions for CO₂ emissions and feedstock consumption for the different blue (B1, B2, B3, B4) and turquoise (T) hydrogen production scenarios considered in this study. Figures corresponding to the direct use of natural gas are also provided for comparison^22,27,29,31^. The B1 case is based on real-world data and current technologies that have been in continuous operation for several years, notably at the Petra Nova Gorgon facility in the United States^22,23^. The carbon dioxide emissions and the amount of methane required as a feedstock for hydrogen production are based on the SMR process, with a CO₂ capture rate of 85% for the SMR and 65% for driving the SMR. Case B1 was used in our previous calculations and is used again in the current analysis for comparison with other blue hydrogen production scenarios^11^. In addition to this B1 case representative of a realistic operating industrial-scale SMR plant, we introduce several other scenarios for potential future blue hydrogen production facilities. The B2 case corresponds to a theoretical conventional natural gas SMR modern blue hydrogen plant with a CO₂ capture amine process, designed to achieve 90% efficiency^29,32^. This high-capture-rate scenario implies an increase in natural gas consumption and a reduction in the excess electricity that could be exported to the grid. This option also implies an increase in hydrogen production operating costs and the total plant cost, which we do not discuss further in our analysis. The B3 case is again a theoretical blue hydrogen production plant that uses natural gas or biomethane. It is based on an oxygen-blown autothermal reformer (ATR) and an optimised monoethanolamine (MEA) CO₂ capture and storage unit, resulting in an overall capture efficiency of 93%^28,29^. The B4 case is a also theoretical low-carbon hydrogen production plant that combines recuperative reforming, pre- and post-combustion capture and hydrogen-rich firing. These elements have been demonstrated individually in existing hydrogen and ammonia production plants but not in an operating plant nor at industrial scale. The key data for this blue hydrogen production plant is based on Equinor studies in Norway and assumes a capture rate of 96% efficiency for the plant^27^. In addition to the above scenarios involving the SMR process, case T, also considers an alternative turquoise hydrogen production scenario from high-temperature methane pyrolysis^31^. This scenario assumes that the pyrolysis of methane is carried out via thermal plasma, resulting in the co-production of solid carbon black. This is based on data from the Monolith Inc. Olive Creek facility. It is also assumed that the electricity used in the plant comes from low-carbon renewable sources. The carbon intensity of this hydrogen production method is calculated using a mass allocation method weighted by the mass of hydrogen and carbon black produced^31^.

**Table 1:** Total carbon dioxide emissions from the plant and from indirect feedstock processing; and natural gas consumption for the different blue (B1, B2, B3, B4) and Turquoise (T) hydrogen production options considered in this study. Numbers for the Turquoise hydrogen scenario are mass weighted with the coproduced carbon black (19%). The natural gas end use numbers are also provided for comparison^22,27,29,31^. Higher heating values (HHV) are provided for comparison with previous work.

scenario	Total feedstock consumption (gCH_4_/MJ_HHV_)^a^	CO_2_ emissions from the plant (gCO_2_/MJ_HHV_)^b^	Feedstock processing (gCO_2_/MJ_HHV_)^c^
B1 – Blue hydrogen Petra Nova Gorgon facility in operation − 65% CCS^22^	31.6	33.1	6.5
B2 – Blue hydrogen theoretical modern SMR facility − 90% CCS^29,32^	26.6	6.99	5.07
B3 – Blue hydrogen theoretical ATR facility − 93% CCS^28,29^	24.7	4.64	4.70
B4 – Blue hydrogen theoretical Equinor − 96% CCS^27^	22.9	6.10	4.36
T – Turquoise hydrogen methane pyrolysis Monolith facility^31^	8.10	0.99	1.54
Natural gas	18.0	49.5	3.70

^a^Includes the direct feedstock consumption to produce hydrogen, to generate the heat and pressure needed for the SMR, and the amount needed to drive the CO_2_ capture.

^b^Includes the direct emission from the SMR process itself, the emission associated with the energy to drive the SMR process, and the emission associated to power the CO_2_ capture process.

^c^Indirect upstream emissions for natural gas processing and transport.

In the case of hydrogen fuel, the leakage rate is an important factor of uncertainty in the overall hydrogen economy. As hydrogen is an indirect climate gas, the leakage rate is a key factor in determining the carbon equivalent intensity of a transition to hydrogen fuel^11,12,15^. Similarly, the leakage rate of methane is critical in determining the climate impact of a blue hydrogen production^22,27,29^. In our calculations, we vary the hydrogen leakage rate from 0.1 to 15% in order to determine the hydrogen climate footprint and we derive methane leakage rate based on the considered hydrogen leakage rate. In contrast to our previous work^11^, a maximum leakage rate is applied to methane (See “Methods”).

**Fig. 1:**
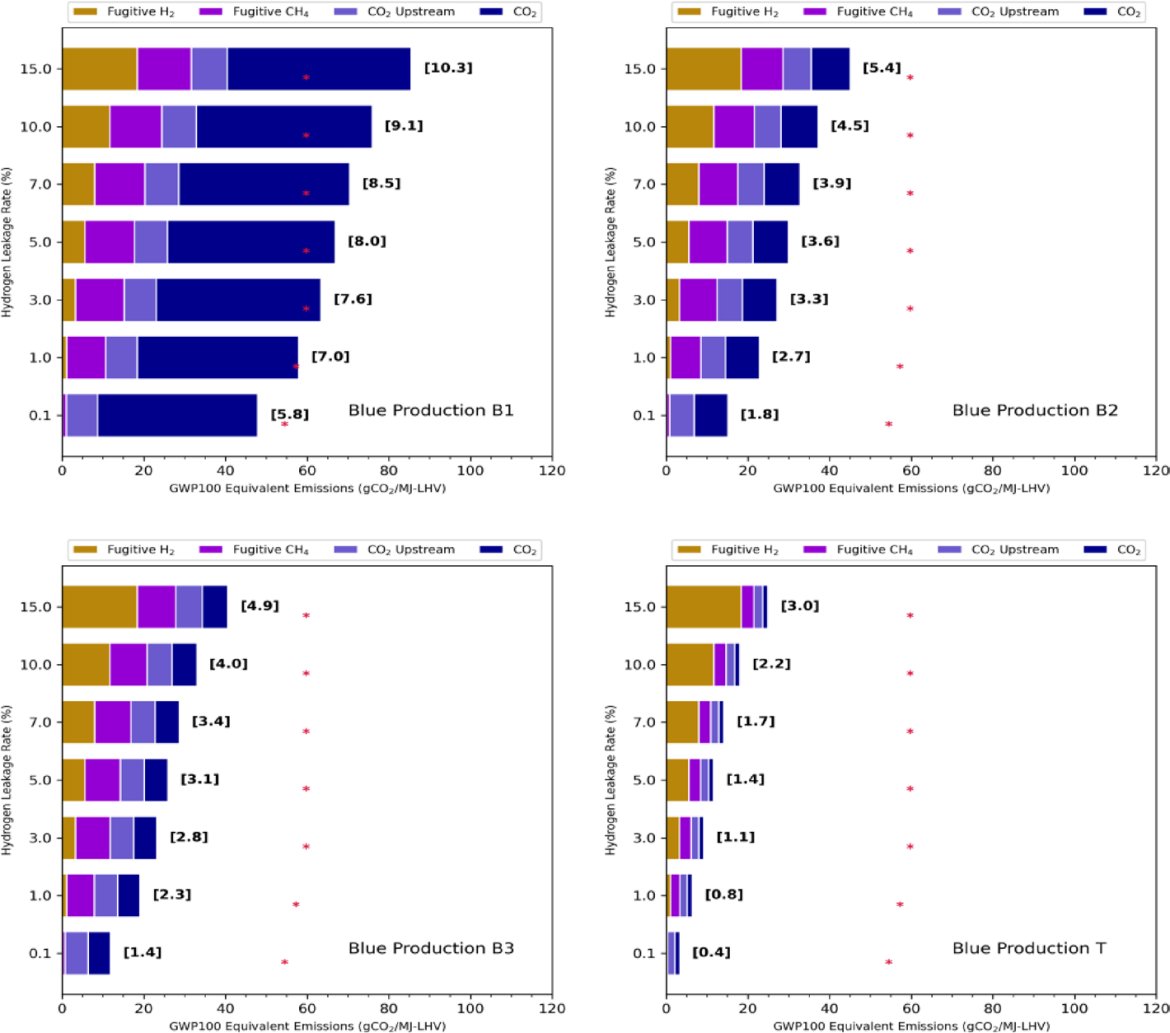
Blue hydrogen carbon footprint. The carbon footprint (gCO_2_eq/MJ_LHV_) of different blue hydrogen production options (B1, B2, B3, T) calculated as a function of the hydrogen leakage rate (%). Values under brackets provide the corresponding footprint in kgCO_2_eq/kgH_2_. The total footprint is decomposed into hydrogen fugitive emissions, methane fugitive emissions, and upstream CO_2_ and SMR process CO_2_ emissions. The corresponding footprint of direct use of natural gas is also shown for comparison (red stars). The equivalent emissions are calculated based on the GWP100 emission metric assuming a 1% methane maximum leakage rate.

**Fig. 2:**
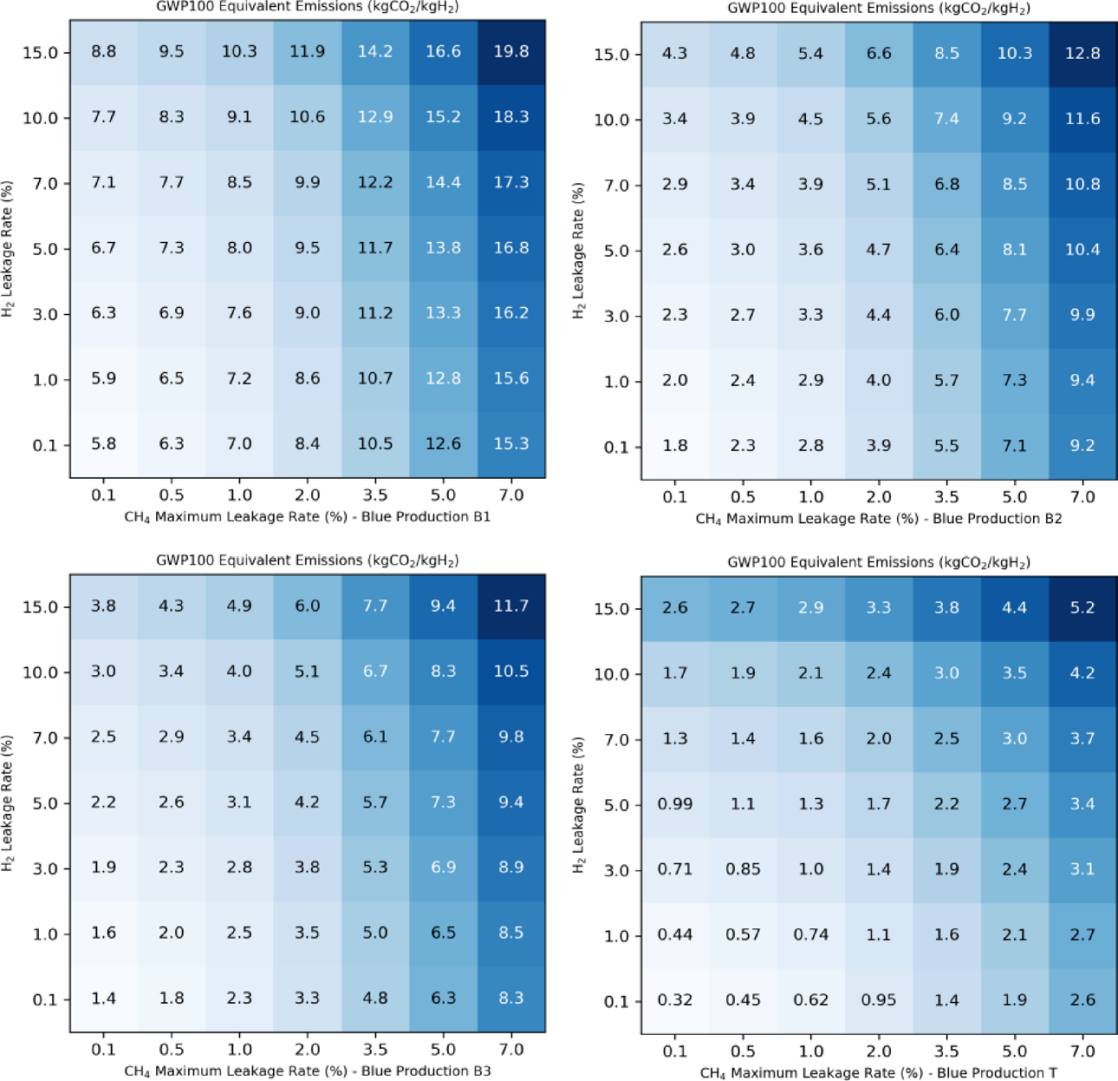
Blue hydrogen carbon footprint. The total carbon footprint (kgCO_2_/kgH_2_) of different blue hydrogen production options (B1, B2, B3, T) calculated as a function of the hydrogen leakage rate (%) and the methane maximum leakage rate (%).The equivalent emissions are calculated based on the GWP100 emission metric.

First, we determine and compare the carbon footprint of the various blue (and turquoise) hydrogen production and distribution methods examined in this study. Figure 1 shows the total hydrogen production footprint for the various blue hydrogen production methods and for varying hydrogen leakage rates. To calculate this total footprint we account for the (1) CO_2_ emissions associated with the reforming process itself, including the energy used for heat and pressure to drive the reforming after incomplete carbon capture, and from the energy used to drive the carbon capture; (2) the upstream CO_2_ emissions associated with producing and transporting natural gas; (3) the fugitive methane emissions during the transport and use of the natural gas; (4) the fugitive hydrogen emissions. The methane and hydrogen CO_2_ equivalent emissions are calculated based on the GWP_100_ emission metric, with a maximum baseline CH_4_ leakage rate of 1% assumed. The carbon footprint of blue (or turquoise) hydrogen production can vary considerably depending on the assumptions made about the production plant. As previously discussed^29^, scenario B1 has a significantly larger climate impact than the other cases, essentially due to a low carbon capture rate and a high CO₂ contribution. In this scenario, the carbon footprint is 7–7.6 kgCO₂eq/kgH₂ for a hydrogen leakage rate of 1–3%. This is 130–170% higher than in scenarios B2, B3 and B4 (not shown). In all cases, fugitive hydrogen emissions represent a sizeable contribution to the total carbon footprint for leakage rates higher than 3%. These results demonstrate that, for high hydrogen leakage rates, this contribution can even dominate the total carbon footprint, except in case B1. A carbon footprint as low as 0.77–1.1 kg CO₂eq/kg H₂ is calculated for turquoise hydrogen for a hydrogen leakage rate of 1–3%. In cases B2, B3 and B4, fugitive methane emissions account for around a third of the climate impact due to the hydrogen production. The carbon footprint associated with the direct use of natural gas is also shown for comparison. In all cases except B1, the carbon footprint is 2–3 times lower for hydrogen production and use. Supplementary Figure S1 compares the total carbon footprint calculated using the GWP100 metric to the footprint derived using the GWP20 metric. Due to the shorter time horizon used to determine the CO_2_ equivalent emissions of hydrogen and methane, we derive a footprint increasing to 9.3–11.0 kgCO_2_eq/kgH_2_ for a 1–3% hydrogen leakage rate in case B1 and a footprint of 4.0-6.1 kgCO_2_eq/kgH_2_ in scenario B2-B3-B4. Figure 2 shows how sensitive the calculated blue hydrogen production footprint is to the chosen maximum methane leakage rate. The results show that the footprint varies significantly with the maximum leakage rate of methane and, in case B1, ranges from 5.8 to 8.8 kgCO_2_eq/kgH_2_ for a small CH_4_ leakage rate of 0.1% to 15-3-19.8 kgCO_2_eq/kgH_2_ for a leakage rate of 7%. For case B2, the carbon footprint decreases significantly and ranges from 1.8 to 4.3 kgCO_2_eq/kgH_2_ for a small CH_4_ leakage rate of 0.1% (0.3–2.6 kgCO2eq/kgH2 for case T). For a very small hydrogen leakage rate of 0.1%, the carbon footprint for cases B1, B2 and B3 is respectively 7.0, 2.8, and 2.3 kgCO_2_eq/kgH_2_ for a 1.0% methane maximum leakage rate, and 10.5, 5.5, and 4.8 kgCO_2_eq/kgH_2_ for a 3.5% methane maximum leakage rate. These results compare very favorably with those of a recent study, which calculated a carbon footprint of 7.0, 2.8 and 2.4 kgCO₂eq/kgH₂ for cases B1, B2 and B3 with a 1% methane leakage rate, and 10.3, 5.7 and 5.0 kgCO₂eq/kgH₂ for a 3.5% methane leakage rate^29^. Our results show that fugitive hydrogen emissions play a minor role for leakage rates below 3% in the case of blue hydrogen B1, but they make a significant contribution to the carbon footprint as leakage rates increase, particularly in the more optimistic blue hydrogen production scenarios B2, B3 and T. In the following, we will present the results for a maximum leakage rate of 1% for methane.

**Fig. 3:**
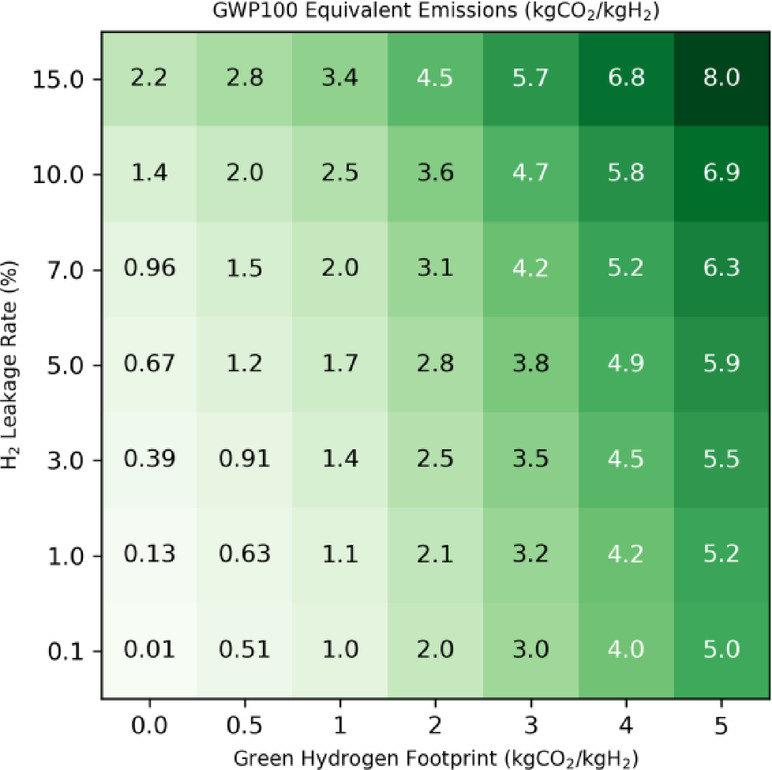
Green hydrogen carbon footprint. The total carbon footprint (kgCO_2_eq/kgH_2_) of green hydrogen as a function of the assumed hydrolysis from renewable electricity footprint (kgCO_2_eq/kgH_2_) and the hydrogen leakage rate (%). The equivalent emissions are calculated based on the GWP100 emission metric.

Green hydrogen is, by definition, produced through electrolysis driven by renewable energies. Its carbon footprint depends on the source and allocation of the renewable electricity and can vary from 0.4 to 0.8 kgCO₂eq/kgH₂ for an offshore wind turbine to 2.1–5.6 kgCO₂eq/kgH₂ for a 2030 clean grid mix^21^. The International Energy Agency’s Net Zero Emissions future scenario assumes a footprint as little as 0.8–0.9 kgCO₂eq/kgH₂ by 2050^20^. For the sake of comparison with blue hydrogen, we also include a production footprint for green hydrogen in our calculations. Figure 3 shows the equivalent CO_2_ emissions calculated as a function of the hydrogen leakage rate and for a green hydrogen production footprint in the range 0 to 5 kgCO_2_eq/kgH_2_. The green hydrogen footprint becomes significant compared to the optimistic blue hydrogen scenarios B2, B3, B4 and is even higher than the turquoise hydrogen production footprint. For our future simulations spanning 2030–2100, we use a baseline green hydrogen production footprint of 1 kgCO_2_eq/kgH_2_, calculated by assuming a 50% mix of production from wind electricity (0.6 kgCO_2_eq/kgH_2_) and from photovoltaic electricity (1.3 kgCO_2_eq/kgH_2_)^20^. This value is also in line with the IEA estimate for 2050^20^. In this case, the footprint including fugitive hydrogen emissions ranges from 1 to 3.4 kgCO_2_eq/kgH_2_ for a hydrogen leakage rate of 0.1–15%. In order to examine the sensitivity of our results to this parameter, we also illustrate a higher green hydrogen production footprint of 3 kgCO_2_eq/kgH_2,_ a value close to the recommended threshold for low-carbon hydrogen^18^. In this case, the footprint including fugitive hydrogen emissions ranges from 3 to 5.7 kgCO_2_eq/kgH_2_ for a hydrogen leakage rate of 0.1–15%.

### Climate benefit of blue hydrogen

**Fig. 4:**
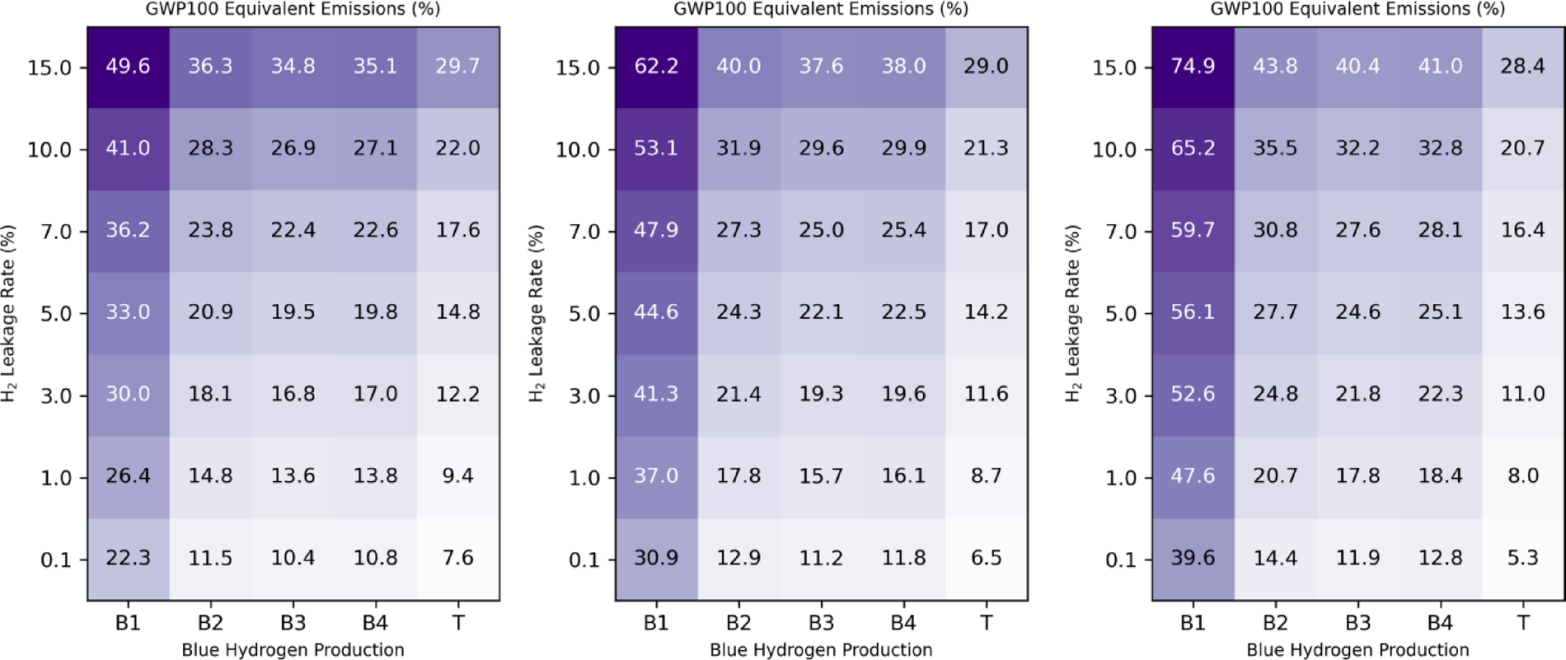
Climate benefit of a global energy transition to hydrogen. Ratio (expressed in %) of CO_2_ equivalent emissions associated with a hydrogen economy to the avoided CO_2_ emissions as a function of the hydrogen leakage rate and the blue hydrogen production option. The emission ratios are calculated assuming 30% (left), 50% (middle) and 70% (right) of blue hydrogen supply. The equivalent emissions are calculated based on the GWP100 emission metric assuming a 1% methane maximum leakage rate and 1 kgCO_2_eq/kgH_2_ footprint for green hydrogen production from hydrolysis.

We then determine the climate benefit of a hydrogen economy by dividing the remaining CO_2_ equivalent emissions from the production and use of hydrogen by the avoided CO_2_ emissions. The equivalent CO_2_ emissions for a hydrogen economy are calculated as the sum of the previously discussed carbon footprint of the green or blue hydrogen production including the hydrogen fugitive emissions. CO_2_ emission abatement is calculated on the basis that 10.9 kgCO_2_ are avoided per kgH_2_ across all energy supply sectors. This value is in line with the CertifHy hydrogen certification scheme^33^ which defines a fossil fuel comparator for hydrogen of 10.92 kgCO_2_eq/kgH_2_. We also account for the direct H_2_ emissions to the atmosphere resulting from fossil fuel combustion (see “Methods”). Figure 4 shows the remaining CO_2_ equivalent emissions for the different blue hydrogen production assumptions, plotted as a function of the hydrogen leakage rate. These figures are calculated using the GWP100 emission metric assuming a maximum methane leakage rate of 1%. We consider a hydrogen mix of green and blue hydrogen with the blue hydrogen fraction Set to 30%, 50% or 70%. The 30% blue hydrogen mix for the case B1 is an update from our previous calculations^11^. In this case, the remaining CO_2_ equivalent emissions are 22% (abatement of 78%) for a H_2_ leakage rate of 0.1%. This value is higher than the 16% calculated in our previous study, primarily due to the inclusion of a green hydrogen production carbon footprint as discussed above. For a high H_2_ leakage rate of 15% the remaining CO_2_ equivalent emissions is about 50%. This value is significantly lower than the 92% previously calculated^11^ Since we now impose a maximum leakage rate of 1% for methane. Using less emitting blue hydrogen production pathways significantly reduces these remaining emissions. With a 1% H_2_ leakage rate the remaining emissions decrease from 26% (abatement of 74%) for case B1 to approximately 14% (abatement of 86%) for the blue hydrogen cases B2, B3 and B4, and to just 9% (91% abatement) for the turquoise hydrogen production. Increasing the proportion of blue hydrogen in the mix significantly reduces the benefits of a hydrogen economy in case B1. With a 1% hydrogen leakage rate the remaining emissions increase from 26 to 37% and 48% as the blue hydrogen fraction increases from 30% to 50 and 70%. However, for the other blue hydrogen scenarios, the remaining emissions increase only slowly with the blue hydrogen fraction in the mix due to their low carbon footprint. Even at 70% blue hydrogen fraction, the remaining emissions generally remain around 20–25% for a 1–3% hydrogen leakage rate (around 30% remaining emissions for a higher leakage rate of 5–7%) for the B2, B3, and B4 cases. This significant climate benefit arises from Switching to a blue hydrogen production plant with a low carbon footprint. In the case of turquoise hydrogen, increasing the fraction in the mix even reduces the remaining emissions further, since the production carbon footprint is lower than that assumed for green hydrogen production. We confirm that hydrogen fugitive emissions significantly reduce the climate benefit at high leakage rates. For a 50% blue hydrogen mix, the remaining emissions increase from approximately 12% at a leakage rate of 0.1% to around 40% at a leakage rate of 15% for cases B2, B3 and B4. The use of a higher green hydrogen footprint of 3 kgCO_2_eq/kgH_2_ significantly reduces the climate benefit (Supplementary Fig. S2). As expected, for a blue hydrogen fraction of 30%, the remaining emissions increase by more than to a factor of 2, in particular when the blue hydrogen production carbon footprint becomes lower than the green hydrogen production footprint (cases B2-T). For case B1, with a 1% hydrogen leakage rate the remaining emissions increase from 39 to 46% and 53% as the blue hydrogen fraction increases from 30% to 50 and 70%.

**Fig. 5:**
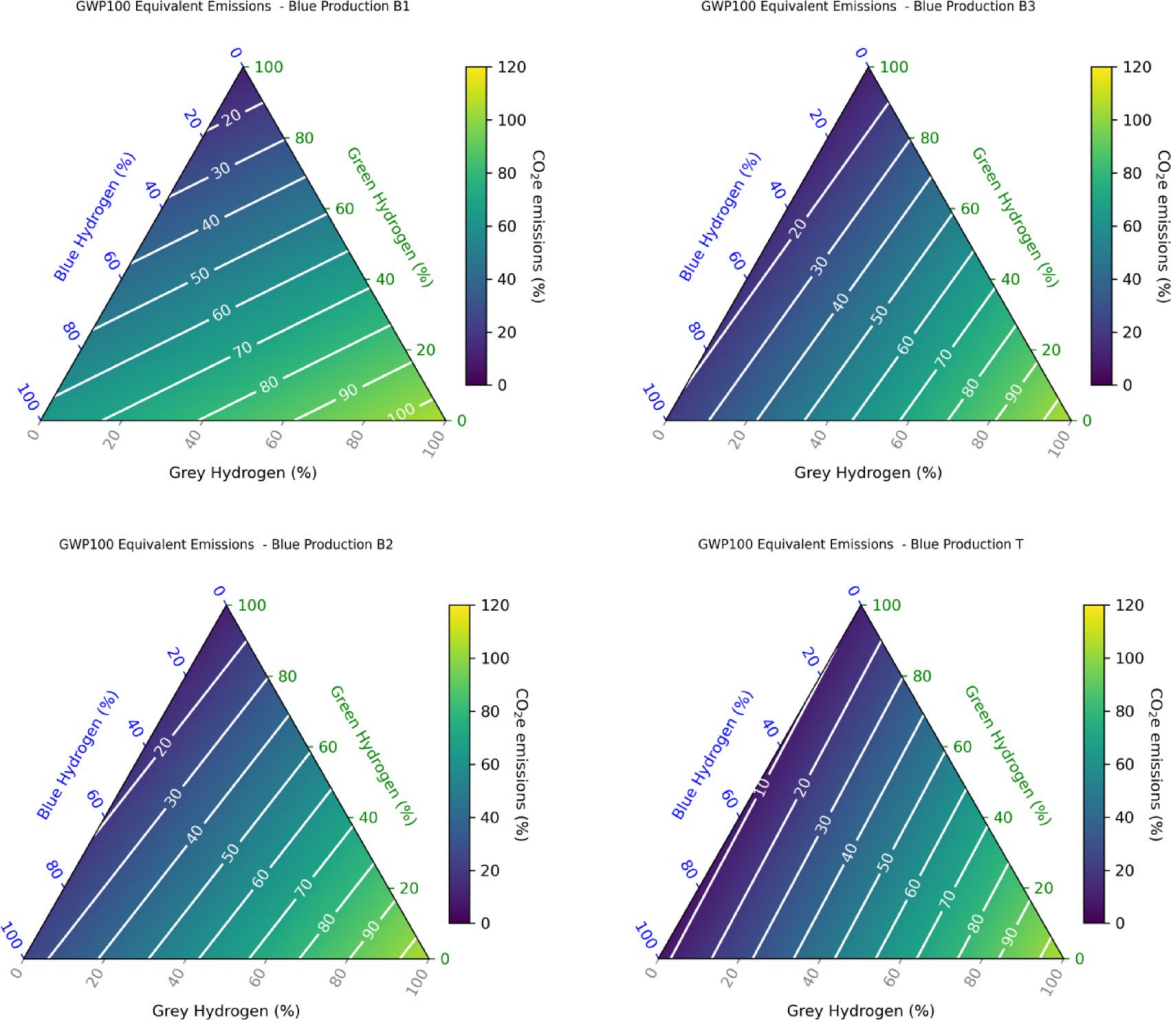
Climate benefit of a global energy transition to hydrogen. Ratio (expressed in %) of CO_2_ equivalent emissions associated with a hydrogen economy to the avoided CO_2_ emissions as a function of the grey, blue or green hydrogen fractions in the mix (%) and for the different blue hydrogen production options (B1, B2, B3, B4, T). The equivalent emissions are calculated based on the GWP100 emission metric assuming a 1% methane maximum leakage rate and 1 kgCO_2_eq/kgH_2_ footprint for green hydrogen production from hydrolysis.

Figure 5 provides a more detailed breakdown of the remaining equivalent CO₂ emissions for the various blue hydrogen scenarios, presented as a function of the grey, blue, and green hydrogen proportions within the energy mix. These results were calculated for a fixed hydrogen leakage of 1%. Remaining emissions are lower than 20% for a green hydrogen fraction greater than 90%, and greater than 100%, thereby inducing a climate penalty for a grey hydrogen fraction greater than approximately 90%. This figure confirms a gradual shift towards remaining emissions of less than 20% for high blue hydrogen fractions in the B2, B3 and B4 (not shown) cases. These remaining emissions are even lower than 10% for turquoise hydrogen (case T), which has a smaller carbon footprint than green hydrogen. This figure clearly shows that the assumed future production method of blue hydrogen has a significant impact on the climate benefit of a hydrogen energy transition. Increasing the green hydrogen carbon footprint to 3 kgCO_2_eq/kgH_2_ shows that the future green hydrogen production method also remains a key parameter to maximize the climate benefit of a hydrogen economy in the future (Supplementary Fig. S3). As expected from the carbon footprints shown previously, in this case, increasing the use of a cleaner blue hydrogen production method (B2-T) in the mix becomes clearly more beneficial to reduce the CO_2_ emissions than the use of green hydrogen.

### Cumulative CO_2_ emissions abatement

We use three different scenarios for the future development of a global hydrogen economy and now examine the climate benefit of the energy transition between 2030 and 2100. These scenarios include the energy demand required to produce the total amount of hydrogen needed to satisfy this demand, calculated based on a hydrogen energy content of 120 MJ/kg, assuming a Low Heating Value (LHV). The scenarios also consider the contributions of grey, blue and green hydrogen to the total. The three scenarios considered in a previous study^11^ are used again in the present work for comparison with our previous results (see “Methods” for a brief description of the different scenarios considered). The HC2017 scenario^34,35^ assumes an increase of the hydrogen end-use demand to reach 650 MtH_2_/year in 2050 across the entire value chain. The more ambitious hydrogen scenario HCKM2021^36^ assumes a maximum hydrogen demand of 783 MtH_2_/yr. Finally, the IEA2021 scenario^37^ assumes a hydrogen demand of 728 MtH_2_/yr in 2050. These future scenarios generally consider 2030 or 2050 as a maximum time-horizon for future hydrogen end-use demand projection. In our base-case calculations, as a conservative approach, the 2050 hydrogen demand is assumed constant for the period 2050–2100 for these three scenarios. Based on these three different hydrogen demand scenarios, we calculate the cumulative CO_2_ abatement arising from the development of a future hydrogen economy over the 2030–2100 period. We calculated the total avoided CO_2_ equivalent emissions assuming an abatement of 10.9 kgCO_2_ per kgH_2_ across all the energy supply sectors^33^. To this value, we add the CO_2_ emissions associated with the hydrogen production, the fugitive methane emissions and the fugitive hydrogen emissions according to the carbon footprint presented above. We assume a maximum methane leakage rate over the entire value chain while varying the hydrogen leakage rate from 0.1 to 15%. The proportions of grey, blue and green hydrogen production in the mix are provided by the different scenarios (see “Methods”). For these calculations we assume the various blue hydrogen production cases (B1, B2, B3, B4 and T) for the entire 2030–2100. As discussed earlier, the B1 blue hydrogen case assumes a lower carbon sequestration rate based on current technologies, whereas the B2 (and B3, B4) scenario considers much higher sequestration rates based on theoretical blue hydrogen production plants that are not currently available. Based on these considerations we introduce an additional scenario labelled B1B2 in which the B1 case is assumed until 2050, after which the B2 case is assumed until 2100, provided the CCS technology is then available. As a sensitivity analysis, we also examine a rather speculative “high-demand” alternative option for hydrogen end-use demand over the period 2050–2100, assuming the trend between 2040 and 2050 remains constant throughout the second half of the 21st century for the three considered scenarios.

**Fig. 6:**
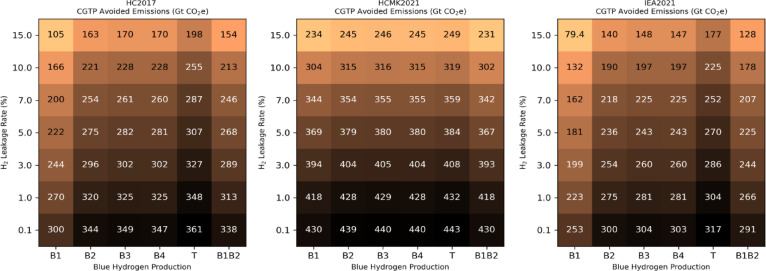
Abatement of CO_2_ emissions calculated based on the CGTP metric. Cumulative CO_2_ equivalent emissions (GtCO_2_eq) abatement over the 2030–2100 period associated with a hydrogen economy as a function of the hydrogen leakage rate. The CO_2_ equivalent emissions are calculated based on the CGTP metric for three scenarios: HC2017, HCMK2021, and IEA2021. The emission abatement is calculated considering different blue hydrogen production options (B1, B2, B3, B4, T, B1B2).

Previous studies have shown that the CGTP metric, which combines sustained and pulse emission responses, is particularly effective in converting emissions of short-lived species such as methane or H_2_ into equivalent cumulative carbon emissions^11,38^. To illustrate the progress towards climate change mitigation associated with a hydrogen economy, we use different hydrogen scenarios and the CGTP metric to calculate the reduction in cumulative carbon emissions between 2030 and 2100. Figure 6 shows the avoided carbon emissions for various blue hydrogen production scenarios in relation to the hydrogen leakage rate. For a low hydrogen leakage rate of 0.1% across the entire value chain and for the B1 blue hydrogen production case, 253–430 GtCO_2_eq are avoided depending on the future scenario. Depending on the scenario, increasing the hydrogen leakage rate to 1% reduces the avoided carbon to between 223 and 418 GtCO₂eq. Increasing the leakage rate to 10% reduces the avoided carbon to 132–304 GtCO₂eq. This value is significantly higher than that calculated in a previous estimate^11^ primarily because our calculations now assume a maximum methane leakage rate of 1%. Improving the carbon footprint of the blue hydrogen production plant increases CO₂ abatement from 270 GtCO₂eq (case B1) to 320–349 GtCO₂eq (cases B2–T) for scenario HC2017 and a 1% hydrogen leakage rate. In the B1B2 case, assuming that low-carbon footprint blue hydrogen production is ready by 2050, the CO_2_ abatement is in the range 266–418 GtCO_2_eq depending on the future scenario and for a 1% hydrogen leakage rate. Previously, it was found that the results obtained with the CGTP were very close to the emission abatements calculated based on the GWP40^11^. For comparison, CO₂ abatement estimates based on other emission metrics and time horizons for methane and hydrogen fugitive emissions are also provided (Supplementary Fig. S4 for GWP100, Fig. S5 for GWP20 and Fig. S6 for GTP100).

**Fig. 7:**
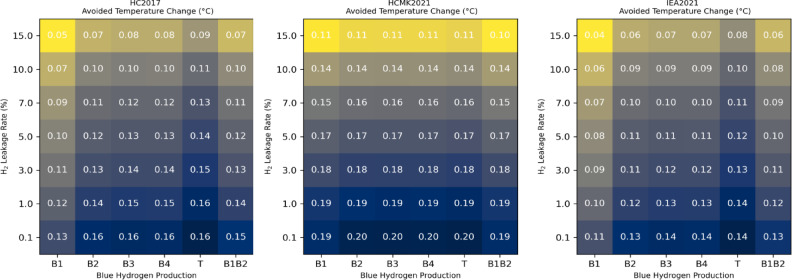
Avoided climate warming in 2100 calculated based on the CGTP metric. Avoided climate warming in 2100 (°C) associated with a hydrogen economy as a function of the hydrogen leakage rate. The avoided warming is calculated based on the CGTP metric and the TCRE (Transient Climate Response to cumulative CO_2_ Emissions) for three scenarios: HC2017, HCMK2021, and IEA2021. The avoided warming is calculated considering the different blue hydrogen production options (B1, B2, B3, B4, T, B1B2).

The amount of global warming per unit of cumulative carbon dioxide emissions is known as the transient climate response to cumulative CO_2_ emissions (TCRE)^39,40^. This TCRE relationship can be used to translate the avoided cumulative CO_2_ emissions associated with a hydrogen economy into an avoided climate warming. The TCRE is estimated to be in the range of 0.27–0.63 °C per 1000 CtGO_2_, with a best estimate of 0.45 °C per 1000 GtCO_2_^41^. Based on this best estimate of the TCRE and on the avoided cumulative CO_2_ emissions depicted in Fig. 6, we can derive the avoided climate warming shown in Fig. 7. The avoided warming at the end of the 21 st century reaches a maximum of 0.19 °C for the best-case scenario HCMK2021 and a hydrogen leakage rate of 1%. For a large hydrogen leakage rate of 10% this warming is reduced to 0.14 °C.

The “high-demand” evolution of the hydrogen end-use demand after 2050 which assumes a constant growth over the second half of the 21st century can be seen as a rather speculative and optimistic alternate option for the development of a hydrogen economy. As a sensitivity test, we examine the abatement of CO_2_ emissions calculated for these alternate scenarios. For this “high-demand” evolution, and for the B1B2 case, the CO_2_ abatement is in the range 395–677 GtCO_2_eq depending on the future scenario and for a 1% hydrogen leakage rate (see Supplementary Figure S7). In this case, the avoided warming at the end of the 21 st century reaches a maximum of 0.30 °C for a hydrogen leakage rate of 1% (see Supplementary Figure S8).

## Discussion

Previous studies have shown the benefit of a future hydrogen economy in terms of reducing CO_2_ emissions^11,17^. The hydrogen leakage rate and the green hydrogen fraction in the mix were identified as key factors in maximizing the climate benefit of this energy transition. These conclusions were based on a very conservative blue hydrogen production hypothesis using current technologies^22,23^. However, other blue hydrogen production hypothesis based on theoretical production plants have however been proposed in order to represent future hydrogen production conditions^27,29^.

In this study, we demonstrate the importance of blue hydrogen production hypotheses in estimating the climate benefit of a future hydrogen economy. The hydrogen leakage rate is an important parameter, particularly in the case of green hydrogen production. Depending on the hydrogen leakage rate, we derive a renewable hydrogen carbon footprint that increases by a factor of 2–3 (1–3.4 kgCO₂eq/kgH₂ for a 1 kgCO₂eq/kgH₂ renewable electricity production footprint, and 2–4.5 kgCO₂eq/kgH₂ for a 2 kgCO₂eq/kgH₂ renewable electricity footprint). In the case of hydrogen production from methane reforming, if done properly, the carbon footprint of blue hydrogen production can be substantially lower than that of natural gas, and only twice as large as green hydrogen, when the production footprint through renewable energy is also considered. For blue hydrogen production from SMR + CCS, we calculate a carbon footprint of 7 kgCO₂eq/kgH₂ for current technologies, decreasing to 2.3–2.7 kgCO₂eq/kgH₂ for the new generation of theoretical SMR power plants for a maximum methane leakage rate of 1%. hydrogen production from methane pyrolysis provides an even lower carbon footprint of 0.8 kgCO₂eq/kgH₂ under the assumption that renewable electricity is used to power the pyrolysis process. Clearly, there is significant room for improvement in the carbon footprint of future hydrogen production from methane, and future technologies will be essential to minimize the carbon footprint of hydrogen produced from methane via SMR or pyrolysis.

We find that the climate benefit of a hydrogen economy is substantial when produced using an efficient blue hydrogen production method that sequesters CO_2_ and minimizes the rate of upstream CH_4_ leakage. Taking these new assumptions about hydrogen production from methane into account, it is clear that a transition to a hydrogen economy would benefit the climate. For an H₂ leakage rate of 1–3%, 75–82% of CO₂ emissions are avoided (GWP₁₀₀-based) for the most advanced SMR power plant facilities (90–92% in the case of methane pyrolysis), even with a high blue hydrogen contribution of 70% to the mix. The hydrogen production pathway from methane SMR or pyrolysis and the CH₄ and H₂ leakage rates appear to be key factors in further mitigating the climate impact of the transition to a hydrogen economy.

Based on several scenarios for the development of a 21st century hydrogen economy, we derive significant CO₂ emission reductions of 266–418 GtCO₂eq over the period 2030–2100 (CGTP-based) for a 1% leakage rate, depending on the future hydrogen development scenario. The CO₂ emission reduction even reaches 395–675 GtCO₂eq in the case of the speculative “high-demand” alternative scenarios. This is a substantial reduction in carbon emissions, given that the remaining carbon budget (with a 50% likelihood of limiting global warming to 2 °C) is 1,110 GtCO₂^42^. Developing a hydrogen economy based on low-carbon hydrogen production could help us to stay within this budget over the course of the century. Minimizing the carbon footprint of blue hydrogen production appears essential to achieving this goal. Additionally, the hydrogen leakage rate, and more importantly, the upstream methane leakage rate, appear to be key parameters in achieving this significant carbon abatement. This cumulative CO₂eq emission abatement can be translated into a reduction in global warming of 0.12–0.19 °C by the end of the century (0.18–0.30 °C for the “high-demand” alternative evolution after 2050), based on the TCRE, for a hydrogen leakage rate lower than 1%. This would be a significant contribution to efforts to keep global surface temperature change below 2 °C.

In this work we do not conduct a Life Cycle Analysis or a cradle-to-grave socio-environmental impact assessment of a hydrogen economy and this type of in-depth assessment is essential^15,30^. Instead, we focus on the climate benefit that could be achieved through the development of a hydrogen economy over the 21st century. We account for CO₂ and methane fugitive emissions associated with different modes of hydrogen production, such as steam reforming of fossil gas or pyrolysis, and we include hydrogen leakages in our calculations. In the case of hydrogen produced by electrolysis, we account for the carbon footprint associated with producing the necessary renewable electricity and investigate how sensitive this number is. The environmental impact of different hydrogen production methods should also consider the impact on water resources and the landscape of the renewable electricity needed to produce green hydrogen. For electrolytic hydrogen to be a viable alternative to blue hydrogen, the carbon intensity of the required power must be very low. Until the electricity grid is deeply decarbonized, there is an opportunity cost associated with using renewable energy to produce hydrogen as opposed to integrating it with the power system^43^. This is beyond the scope of the present study. Similarly, cost issues associated with the different hydrogen production modes are not considered in this study^44^. These costs could differ significantly for the blue hydrogen power plants considered in our analysis. Although clean hydrogen can be used directly in various sectors for deep decarbonisation, its relatively low energy density and high production costs have raised doubts as to whether developing clean hydrogen is worthwhile^8^. Direct hydrogen technologies always increase the cost of the energy system^45^. Even with CCS, emissions from gas- or coal-based hydrogen production systems could be substantial, and the cost of CCS is often higher than assumed. The carbon avoidance costs for high capture rates are significant^46^. If all the external costs of each energy carrier are included, hydrogen of any color could become economically competitive in any sector of the energy system. The future success of hydrogen depends heavily on technological development and resulting cost reductions, as well as future priorities and the corresponding policy framework^47^. Furthermore, this work considers the climate benefit of hydrogen across the entire value chain. As discussed in other studies, the leakage rate and carbon footprint of hydrogen production can differ significantly across various activity sectors^15,48^. This study presents a framework that could be applied separately to various specific sectors in future studies.

## Methods

### Climate metrics

The various climate metrics used in this study to derive the climate impact of fugitive hydrogen and methane emissions have been recalculated as a function of the time horizon considered and presented in a previous analysis^11^. These metrics were calculated based on simulation data from the global chemistry-climate model GFDL-AM4.1^49^. In contrast to our previous study^11^, our current analysis focuses on the impact of the blue hydrogen production scenarios and the results obtained for the GWP metric at a 100-yr time horizon and the CGTP. Table S1 shows the key metrics used in this study, based on our previous work^11^, for both hydrogen and methane at the 100-yr and 20-yr time horizons. The metrics have not been recalculated for comparison with our previous results and are very much in line with and within the uncertainty range of a recent intercomparison of global models which gives a GWP_100_ for hydrogen of 11.6 ± 2.8 and a GWP_20_ of 37.3 ± 15.1^12^.

### Future hydrogen emission scenarios

For comparison with our previous work^11^, we base our analysis on the scenarios developed by the hydrogen Council and the International Energy Agency for the worldwide hydrogen energy transition. These scenarios are briefly summarized below and the reader is referred to the full reports for a more detailed overview of the underlying economic and energy assumptions. The reference case scenario used in the current study for a worldwide energy transition is based on the scenario developed by the hydrogen Council as a roadmap for long-term hydrogen deployment^34^. This scenario (referred to here as HC2017) analyses the potential use of hydrogen in the various sectors: transportation, industrial energy, building heating and power, industry feedstock and power generation, and assumes an almost eightfold increase in hydrogen demand in 2050 compared to today. According to this scenario the global energy supply of hydrogen is projected to increase to 14 EJ (3 889 TWh) in 2030; 28 EJ (7 778 TWh) in 2040; and 78 EJ (21 667 TWh) in 2050. Based on the hydrogen Lower Heating Value (LHV) of 120 MJ/kg we derive a demand of 117 MtH_2_/yr in 2030; 233 MtH_2_/yr in 2040; and 650Mt H_2_/yr in 2050. In addition to this total energy supply, this scenario also assumes a supply of 33% grey hydrogen, 33% blue, and 34% green in 2030; a phase-out of grey hydrogen by 2040 with 50% blue and 50% green hydrogen; and 30% blue and 70% green hydrogen in 2050^35^. For the year 2050, the HC2017 scenario results in an annual abatement of 6 GtCO_2_. As a rather conservative scenario, for our base case simulation, we assume the 2050 values for the total hydrogen end-use demand and the green versus blue hydrogen mix over the 2050–2100 period. Over the whole 2030–2100 period, the HC2017 scenario results in a cumulative CO_2_ emission abatement of 331 GtCO_2_. As a sensitivity experiment, we also examine a more speculative “high-demand” alternative scenario for hydrogen end-use demand over the period 2050–2100, assuming the trend between 2040 and 2050 remains constant throughout the second half of the 21st century. In this “high-demand” case, the total hydrogen demand increases and reaches a maximum of 2735 MtH_2_/yr (91 167 TWh) in 2100 for this scenario HC2017. In addition to this reference scenario HC2017, two other scenarios for the future development of the worldwide hydrogen economy are used. A more ambitious hydrogen scenario with hydrogen reaching 22% of the global energy demand in 2050 has been developed by the hydrogen Council and McKinsey & Company^36^. According to this scenario (referred to here as HCMK2021), the hydrogen worldwide end-use demand increases from 20 EJ (5 555 TWh) in 2030; 55 EJ (15 278 TWh) in 2040; to 94 EJ (26 111 TWh) in 2050. This corresponds to 167, 458, and 783 MtH_2_/yr in 2030, 2040 and 2050, respectively. This scenario further assumes 50%, 30% and 20% of grey, blue, and green hydrogen, respectively, in 2030; 5%, 40%, and 55%, respectively in 2040; and 100% green hydrogen in 2050. The HCMK2021 scenario results in an annual CO_2_ emission abatement of 7 GtCO_2_/yr in 2050. If we further assume for our base case simulation that the 2050 hydrogen end-use demand and hydrogen mix are constant for the following decades, over the whole 2030–2100 period, the HCMK2021 scenario results in a cumulative CO_2_ emission abatement of 417 GtCO_2_. We also examine a “high-demand” alternative scenario for hydrogen end-use demand over the period 2050–2100, assuming the trend between 2040 and 2050 remains constant throughout the second half of the 21st century. In this “high-demand” case, the total hydrogen demand reaches a maximum of 2408 MtH_2_/yr (80 267 TWh) in 2100. We also consider the scenario proposed under the “Net Zero by 2050” roadmap developed by the International Energy Agency (hereafter referred to as IEA2021) which assumes a future hydrogen demand of 25.4 EJ (7067 TWh) or 212 MtH_2_/yr in 2030; 46.8 EJ (13 000 TWh) or 390 MtH_2_/yr in 2040; and 63.4 EJ (17 600 TWh) or 528 MtH_2_/yr in 2050^37^. This scenario also assumes 30%, 32% and 38% of grey, blue, and green hydrogen, respectively, in 2030; 9%, 38%, and 53%, respectively in 2040. In 2050, this scenario still assumes a significant proportion of blue hydrogen with 1% of grey, 37% of blue, and 62% of green hydrogen. The IEA2021 scenario results in an annual CO_2_ emission abatement of 5.7 GtCO_2_/yr in 2050. If we further assume for our base case that the 2050 values are constant for the following decades, over the entire 2030–2100 period, the IEA2021 scenario results in a cumulative CO_2_ emission abatement of 353 GtCO_2_. A “high-demand” alternative scenario assuming the trend between 2040 and 2050 remains constant throughout the second half of the 21st century is also considered yielding a maximum H_2_ end-use demand of 1218 MtH_2_/yr (40 600 TWh) in 2100.

### hydrogen and methane leakage rates

The equivalent CO_2_ emissions, at a given time horizon, from methane leakage during the blue hydrogen production are calculated by multiplying the fugitive methane emissions by the corresponding methane climate metric. The hydrogen leakage rate fl_*H2*_ is varied in our calculations from 0.1 to 15% in order to calculate the hydrogen climate footprint and we derive fl_*CH4*_ based on the considered fl_*H2*_ as described in an previous study with a major update^11^. We assume a volumetric leakage ratio between fl_*H2*_ and fl_*CH4*_ of 4 for a laminar flow. For our analysis, we are interested by the mass of the leakage and this ratio must be divided by the density ratio of CH_4_ to H_2_, giving fl_*H2*_/fl_*CH4*_ = 0.5^50^. The methane fugitive emissions are corrected in order to account for the fact that hydrogen is used to replace natural gas use and therefore a fraction of the CH_4_ fugitive emissions would hence also be released to the atmosphere under the use of fossil fuel natural gas use^51^. The methane leakage rate fl_*CH4*_ is calculated to be 0.54 fl_*H2*_ for grey hydrogen and 0.82 fl_*H2*_ for blue hydrogen^11^. This relationship between methane and hydrogen leakage rates are likely to hold only if hydrogen and natural gas are produced and transported with very well managed and reduced leakages in a future energy system. This relationship will not hold for high hydrogen leakage rates or in the case of liquid hydrogen for instance. Therefore, in addition to this relationship we also impose a maximum value on the methane leakage rate and vary this maximum value fl_*CH4,Max*_ in our calculations. We assume a reference value for fl_*CH4,Max*_ of 1% and investigate the sensitivity to this value by varying fl_*CH4,Max*_ from 0.1 to 7%, bearing in mind that the total methane leakage rate fl_*CH4*_ has recently been estimated to be 3.5%^22^. To account for their leakage, the production of hydrogen and methane is increased accordingly to meet the energy needs described in the scenario.

Incomplete combustion of fossil fuels produces CO, which can undergo the water-gas shift reaction to produce hydrogen^52,53^. Reducing fossil fuel emissions by developing a hydrogen economy will reduce these anthropogenic hydrogen emissions from incomplete combustion. hydrogen emitted to the atmosphere from incomplete combustion of fossil fuels has been estimated to be 9.3 Mt/yr over the period 2010-2019^53^. Over the same period, total CO_2_ emissions from fossil fuel combustion are estimated to be 34.5 GtCO_2_/yr^54^. This gives a ratio of 9.3/34.5 MtH_2_/GtCO_2_. We account for this effect by reducing the emission of hydrogen to the atmosphere by 0.27 MtH_2_ per GtCO_2_ abated, by multiplying these emissions by the corresponding H_2_ metric, and add these CO_2_ equivalent emissions to the abated CO_2_ emissions. In the HC2017 scenario, the CO_2_ abatement of 6 GtCO_2_/yr in 2050 reduces the hydrogen emission from incomplete combustion by 1.6 MtH_2_. This figure is to be compared to the fugitive emissions of 6.5 MtH_2_ for a 1% hydrogen leakage rate (1% of 650 MtH_2_/yr).

## Supplementary Information

Below is the link to the electronic supplementary material.

Supplementary Material 1

## Data Availability

The Fortran codes used to calculate the metrics and the metrics data are available from the corresponding author upon reasonable request.

## Code availability

The Python codes used for calculating, analysing and plotting the hydrogen equivalent emissions are available at 10.5281/zenodo.7244779.
