# Supplementary material for: The climate benefit of a greener blue hydrogen
Source: Sci Rep. 2025 Sep 29;15:33409. doi: 10.1038/s41598-025-18765-6 (PMC12479917; doi:10.1038/s41598-025-18765-6)
Supplement: Supplementary file 1 — Supplementary Material 1 [file 41598_2025_18765_MOESM1_ESM.pdf]

The climate benefit of a greener blue hydrogen

Supplementary Material

Didier Hauglustaine

Laboratoire des Sciences du Climat et de l’Environnement (LSCE), CEA-CNRS-UVSQ, Gif-sur-Yvette, France.
E-mail: didier.hauglustaine@lsce.ipsl.fr

Table S1. GWP, GTP, and CGTP metrics for hydrogen and methane at 20- and 100-yr time-horizons used in this study[11]. Metrics are unitless by definition except for CGTP (yr). Uncertainties are 90% confidence levels.

	H_2 (20-yr)	H_2 (100-yr)	CH_4 (20-yr)	CH_4 (100-yr)
GWP	40.1 ± 24.1	12.8 ± 5.2	85.9 ± 27.5	30.9 ± 12.0
GTP	17.8 ± 23.3	2.3 ± 1.5	58.9 ± 23.6	5.8 ± 4.8
CGTP	852 ± 596	1579 ± 1153	1738 ± 626	3786 ± 1628

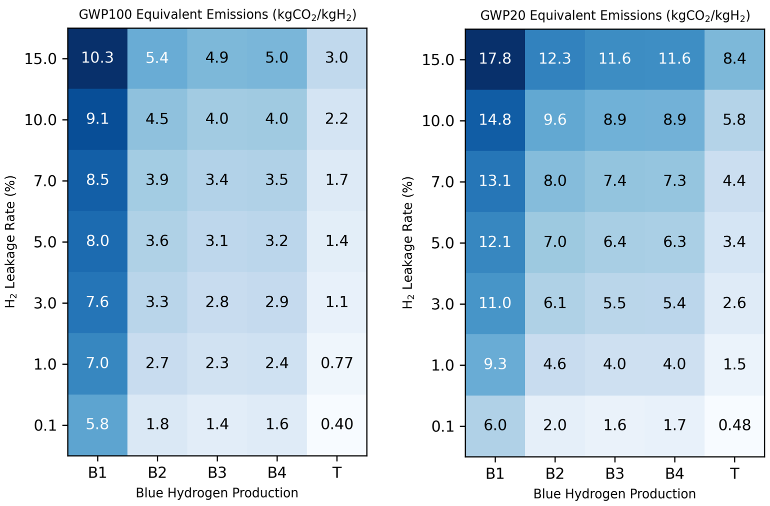

Figure S1. Blue hydrogen carbon footprint. The total carbon footprint ($kgCO_2eq/kgH_2$) of different blue hydrogen production options (B1, B2, B3, B4, T) calculated as a function of the hydrogen leakage rate (%). The equivalent emissions are calculated based on the (left) GWP100 or (right) GWP20 emission metric assuming a 1% methane maximum leakage rate.

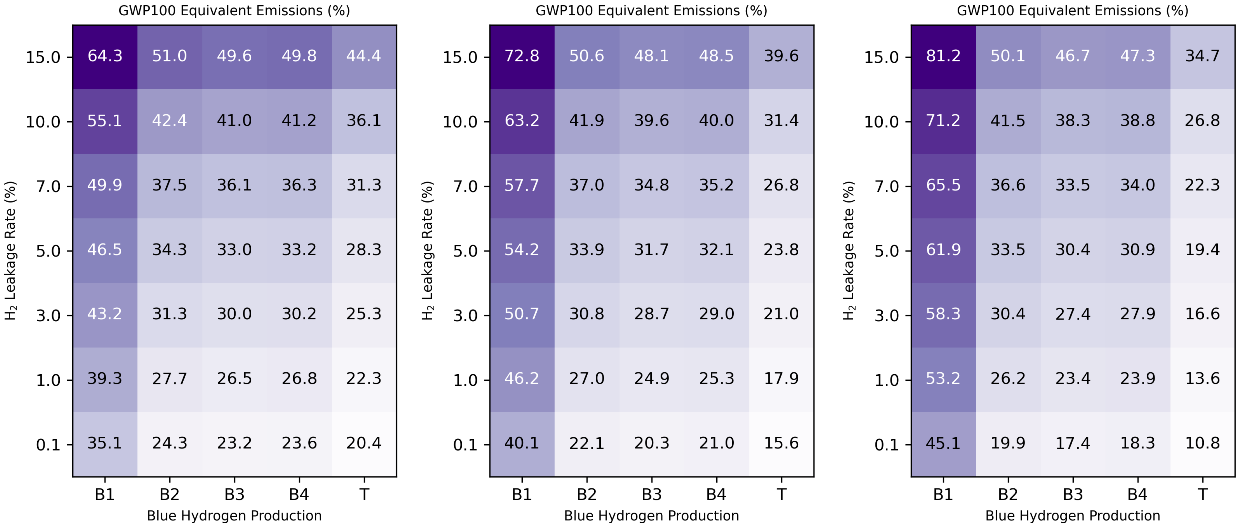

Figure S2. Climate benefit of a global energy transition to hydrogen. Ratio (expressed in %) of CO_2 equivalent emissions associated with a hydrogen economy to the avoided CO_2 emissions as a function of the hydrogen leakage rate and the blue hydrogen production option. The emission ratios are calculated assuming assuming 30% (left), 50% (middle) and 70% (right) of blue hydrogen supply. The equivalent emissions are calculated based on the GWP100 emission metric assuming a 1% methane maximum leakage rate and 3 $kgCO_2eq/kgH_2$ footprint for green hydrogen production from hydrolysis.

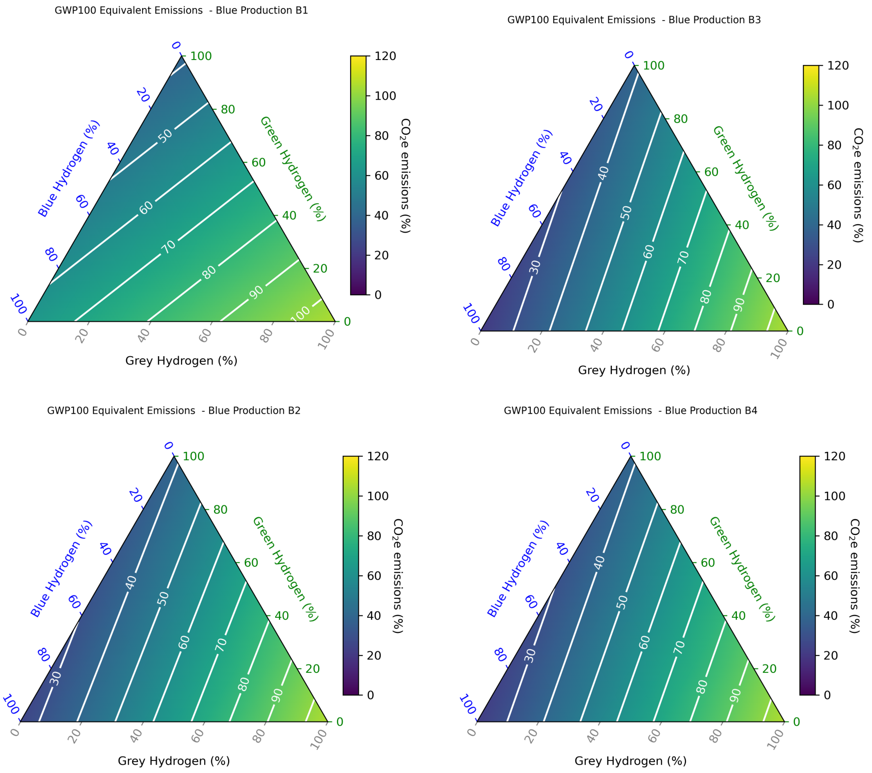

Figure S3. Climate benefit of a global energy transition to hydrogen. Ratio (expressed in %) of CO_2 equivalent emissions associated with a hydrogen economy to the avoided CO_2 emissions as a function of the grey, blue or green hydrogen fractions in the mix (%) and for the different blue hydrogen production options (B1, B2, B3, B4, T). The equivalent emissions are calculated based on the GWP100 emission metric assuming a 1% methane maximum leakage rate and 3 $kgCO_2eq/kgH_2$ footprint for green hydrogen production from hydrolysis.

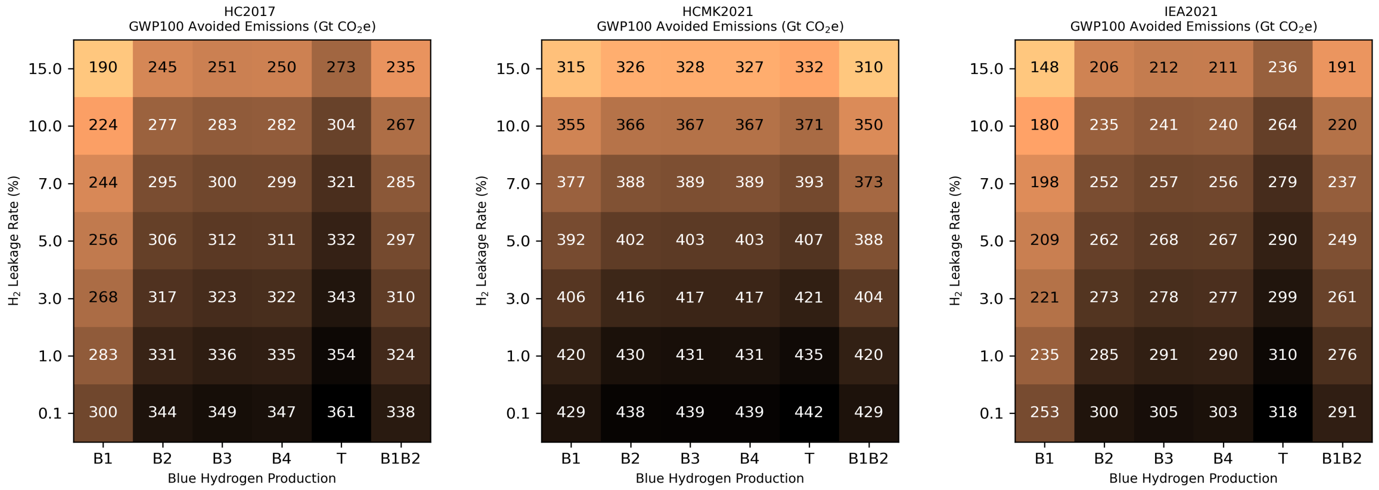

Figure S4. Abatement of CO_2 emissions calculated based on the GWP100 metric. Cumulative CO_2 equivalent emissions ($GtCO_2eq$) abatement over the 2030-2100 period associated with a hydrogen economy as a function of the hydrogen leakage rate. The CO_2 equivalent emissions are calculated based on the GWP100 metric for three scenarios: HC2017, HCMK2021, and IEA2021. The emission abatement is calculated considering the different blue hydrogen production options (B1, B2, B3, B4, T, B1B2).

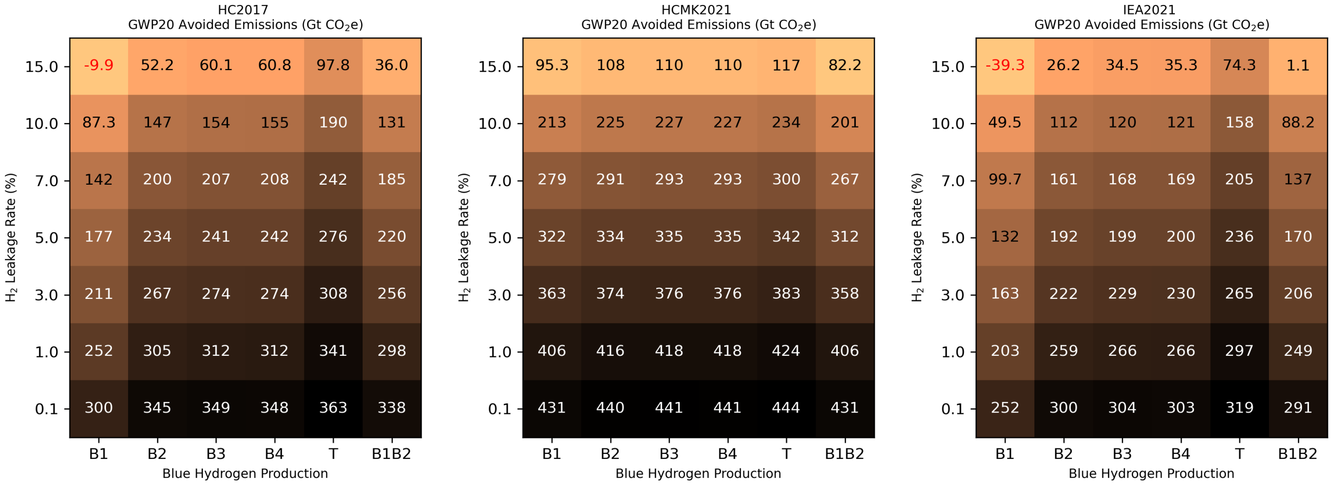

Figure S5. Abatement of CO_2 emissions calculated based on the GWP20 metric. Cumulative CO_2 equivalent emissions ($GtCO_2eq$) abatement over the 2030-2100 period associated with a hydrogen economy as a function of the hydrogen leakage rate. The CO_2 equivalent emissions are calculated based on the GWP20 metric for three scenarios: HC2017, HCMK2021, and IEA2021. The emission abatement is calculated considering the different blue hydrogen production options (B1, B2, B3, B4, T, B1B2).

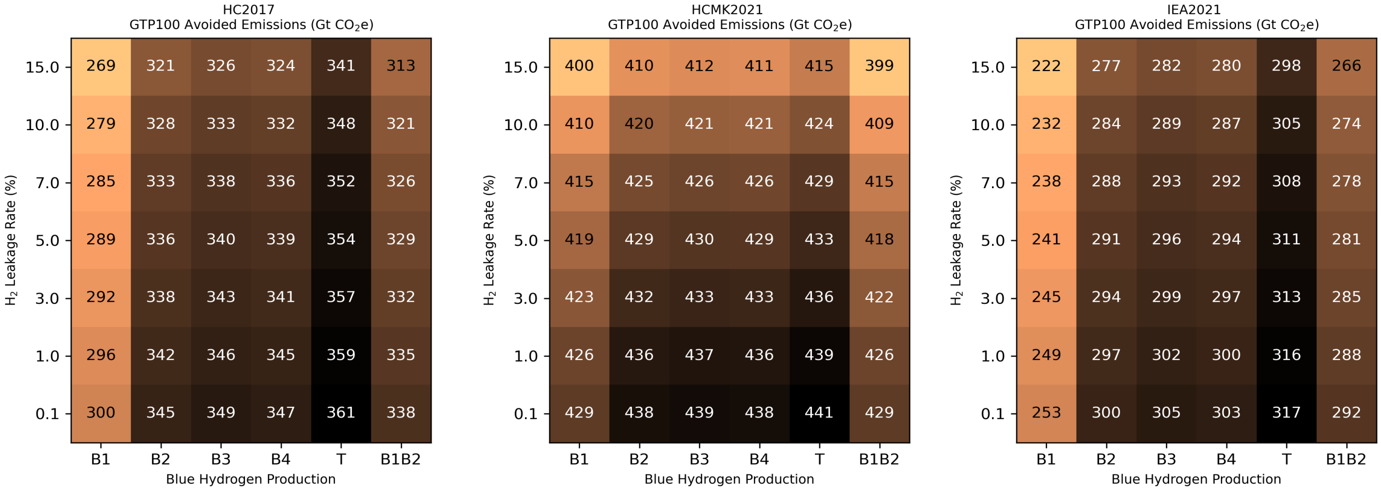

Figure S6. Abatement of CO_2 emissions calculated based on the GTP100 metric. Cumulative CO_2 equivalent emissions ($GtCO_2eq$) abatement over the 2030-2100 period associated with a hydrogen economy as a function of the hydrogen leakage rate. The CO_2 equivalent emissions are calculated based on the GTP100 metric for three scenarios: HC2017, HCMK2021, and IEA2021. The emission abatement is calculated considering different blue hydrogen production options (B1, B2, B3, B4, T, B1B2).

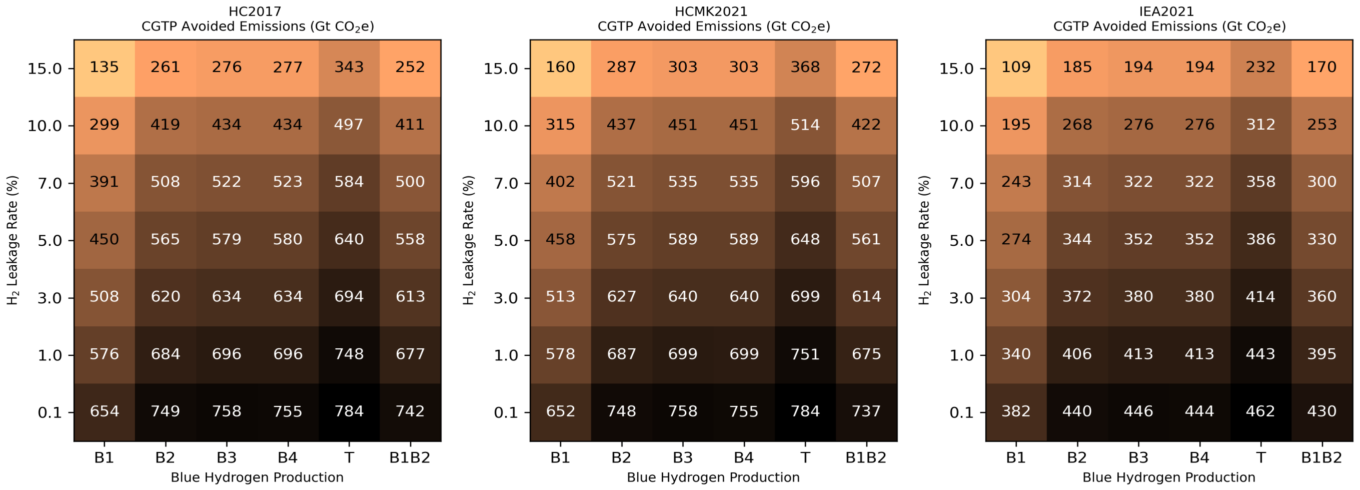

Figure S7. Abatement of CO_2 emissions calculated based on the CGTP metric. Cumulative CO_2 equivalent emissions ($GtCO_2eq$) abatement over the 2030-2100 period associated with a hydrogen economy as a function of the hydrogen leakage rate. The CO_2 equivalent emissions are calculated based on the CGTP metric for three scenarios: HC2017, HCMK2021, and IEA2021. The emission abatement is calculated considering different blue hydrogen production options (B1, B2, B3, B4, T, B1B2). The alternative scenarios assuming a "high H_2 demand" after 2050 are considered here.

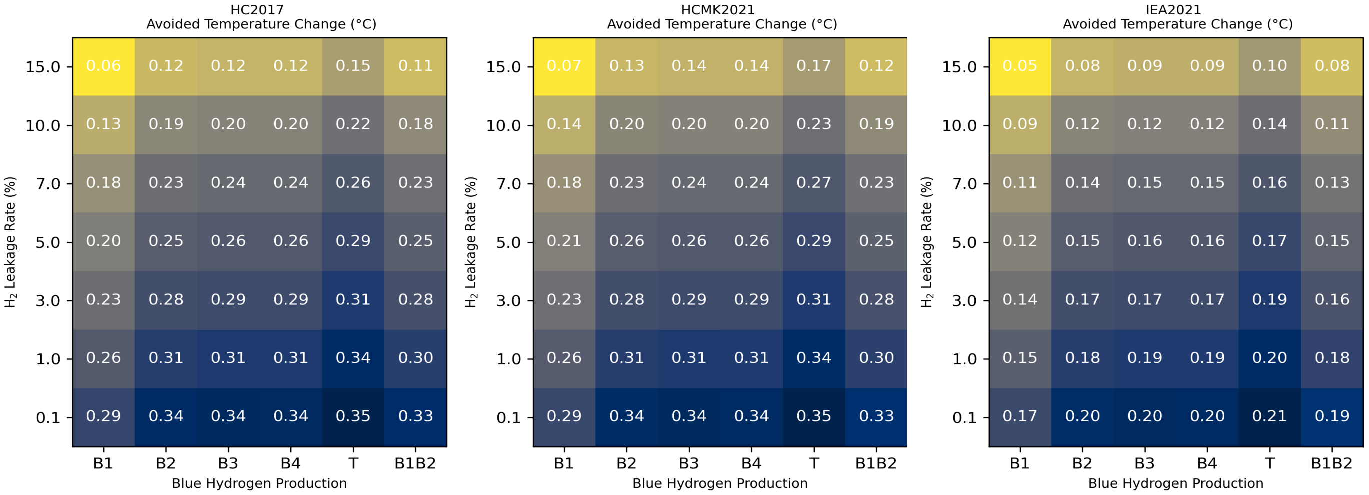

Figure S8. Avoided climate warming in 2100 calculated based on the CGTP metric. Avoided climate warming in 2100 (°C) associated with a hydrogen economy as a function of the hydrogen leakage rate. The avoided warming is calculated based on the CGTP metric and the TCRE (Transient Climate Response to cumulative CO_2 Emissions) for three scenarios: HC2017, HCMK2021, and IEA2021. The avoided warming is calculated considering the different blue hydrogen production options (B1, B2, B3, B4, T, B1B2). The alternative scenarios assuming a "high H_2 demand" after 2050 are considered here.
